# Clinical Spectrum, Diagnostic Characteristics, and Treatment Outcomes of Neurobrucellosis: A Narrative Synthesis of Individual Patient Data From Global Literature

**DOI:** 10.7759/cureus.111587

**Published:** 2026-06-26

**Authors:** Farah Ismail, Ammad Abid, Fiza Ismail, Muhammad Bin Adnan, Nusrat Fatima, Muzafar Mansoor, Abdul Rehman, Haysum Khan, Faiqa I Khan, Hasan Saeed, Haseeb Mehmood Qadri

**Affiliations:** 1 General Surgery, Mayo Hospital, Lahore, PAK; 2 Medicine, Mayo Hospital, Lahore, PAK; 3 Neurological Surgery, Punjab Institute of Neurosciences, Lahore, PAK; 4 General Surgery, Lahore General Hospital, Lahore, PAK; 5 Community Medicine, CMH Lahore Medical College and Institute of Dentistry, Lahore, PAK; 6 Respiratory Medicine, Portsmouth Hospitals University NHS Trust, Portsmouth, GBR; 7 Internal Medicine, Allama Iqbal Medical College, Lahore, PAK; 8 Clinical Research, Shalamar Medical and Dental College, Lahore, PAK; 9 Neurological Surgery, Shifa College of Medicine, Shifa Tameer-e-Millat University, Islamabad, PAK; 10 Histopathology, Shifa International Hospitals, Islamabad, PAK

**Keywords:** agglutination tests, bacterial zoonoses, brain, brucella, brucellosis, neurobrucellosis, review, seizures, zoonoses, anti-infective agents

## Abstract

Neurobrucellosis is a rare and serious complication of brucellosis that can present with a broad spectrum of neurological symptoms, making early detection challenging. The objective of this study was to comprehensively analyze data on neurobrucellosis to characterize its clinical manifestations, diagnostic approaches, and management strategies. This narrative review consolidates the available literature on all cases of neurobrucellosis published between inception and November 30, 2025, using the PubMed Central and Google Scholar databases. The data were stratified using predefined keyword combinations and Boolean operators. All case reports, case series, and original articles with confirmed cases of neurobrucellosis with intracranial involvement were included. After detailed scrutiny of the existing literature, a total of 23 articles were identified with data on 34 patients. Among the 34 patients, 22 (64.70%) were males, and 12 (35.29%) were females. The mean age was 35.7 ± 19.45 years. The most common presenting symptoms were headache in 23 (67.64%), fever in 16 (47.05%), and vomiting in 11 (32.35%) patients. On neurological examination, generalized weakness was present in eight (23.50%) patients, seizures in eight (23.50%) patients, and hearing loss in eight (23.50%) patients. The preferred imaging modality was MRI, which demonstrated hypointense multiloculated lesions on T1-weighted images in 11 (32.35%) patients and hyperintense lesions on T2-weighted images in eight (23.52%) patients. The most frequently involved brain region was the frontal lobe in 11 (32.35%) patients. Serological testing was confirmatory for diagnosis in 30 (88.23%) patients, with positivity on the serum agglutination test. CSF analysis showed elevated protein in 13 (38.23%) patients, leukocytosis in 10 (29.41%) patients, and culture positivity in four (11.76%) patients. The most common species was *Brucella melitensis*, which was identified in six (17.64%) patients. The average time to diagnosis was 8.83 ± 6.80 months. Approximately 21 (61.76%) patients received medical therapy alone, and 12 (35.29%) underwent combined medical and surgical management. Neurobrucellosis often presents with nonspecific prodromal symptoms, making early diagnosis challenging. Imaging commonly reveals frontal lobe involvement with characteristic hypointense multiloculated lesions on T1-weighted MRI and hyperintense signals on T2-weighted MRI. Diagnosis relies on positive serological testing, particularly serum agglutination tests, in conjunction with clinical and radiological findings. Optimal outcomes require a tailored multimodal management approach incorporating appropriate antimicrobial therapy with surgical intervention when indicated.

## Introduction and background

With an estimated annual global burden of over two million cases, brucellosis is one of the most prevalent zoonotic diseases [1]. It remains endemic in regions of Asia, Africa, South America, Central America, the Mediterranean Basin, and the Middle East [2]. The two main routes of transmission are ingestion of unpasteurized dairy products and close contact with infected animals [3,4]. Brucellosis is a multisystem disease that can cause a wide range of clinical manifestations, from fever and myalgia to neurological deficits and visceromegaly [2,5].

The diverse clinical presentation of neurobrucellosis makes clinical diagnosis challenging. The most widely used serological test for confirmation of human brucellosis is the Wright test, which detects elevated IgG and IgM titers [6]. In addition, enzyme-linked immunosorbent assay and the Rose Bengal test may also be used. Tissue culture and PCR may also be used but have low sensitivity and specificity as well as being time-consuming [6,7].

Despite being rare, neurobrucellosis is one of the most severe complications, contributing significantly to morbidity and mortality. It can present with a variety of neurological manifestations, including meningoencephalitis, cranial neuropathies, intracranial hypertension, myelitis, stroke-like events, and brain abscess formation. Its variable presentation, along with inconsistent laboratory and imaging findings, contributes to delays in diagnosis and treatment [1,7]. Despite its clinical significance, neurobrucellosis lacks standardized diagnostic or treatment guidelines, resulting in a knowledge gap that needs to be addressed.

Existing evidence is limited, resulting in fragmented knowledge regarding its clinical spectrum, diagnostic challenges, optimal treatment strategies, and outcomes. This lack of consolidated data often leads to delayed diagnosis and suboptimal management due to the nonspecific presentation and variable radiological findings. By identifying and synthesizing all reported cases of intracranial neurobrucellosis, this study aimed to provide a comprehensive overview of its clinical manifestations, diagnostic approaches, therapeutic regimens, and prognostic factors. Understanding the clinical patterns in reported cases can aid clinicians in earlier diagnosis and facilitate prompt and effective treatment of this covert disease.

## Review

Methodology

A narrative review was conducted to consolidate data on the clinical presentation, diagnosis, and management of neurobrucellosis.

Search Strategy

Data for this narrative review were collected using the PubMed Central and Google Scholar databases. Studies published from inception until November 30, 2025, were included. The search was conducted using Boolean operators (“AND” and “OR”) with the following keywords: “Brucella”, “neurobrucellosis”, “intracranial brucellosis”, “cerebral brucellosis”, “brain infection”, “intracranial infections”, “human brucellosis”, “neurology”, “neurosurgery and brucellosis”, “CNS brucellosis”, “intracranial Brucella”, “meningitis”, “CNS infections”, and “brain abscess”.

Inclusion Criteria

All case reports, case series, and original articles describing neurological manifestations and confirmed cases of neurobrucellosis were included. Articles in English, as well as non-English studies with English translations, were also included.

Exclusion Criteria

All comments, animal and cadaveric studies, short communications, and data describing brucellosis without neurological manifestations were excluded. Additionally, articles with broken links, unavailable complete translated versions, or inaccessible full texts were not included.

Study Selection

The initial search using the specified keywords yielded 467 articles from PubMed and 5,170 articles from Google Scholar. After removing duplicates and applying the inclusion and exclusion criteria, a final total of 23 articles were included, comprising 34 patients. These included 21 case reports describing 21 patients and two case series describing 13 patients, resulting in an overall sample of 34 patients. The studies included are listed in Table 1.

**Table 1:** Details of included studies

Study	Study title	Study type	Age/gender (M/F)	Country
Ayala-Gaytán et al. [8]	*Brucella melitensis*cerebellar abscess	Case report	19 Y/M	Mexico
Kalelioğlu et al. [9]	Brain abscess caused by *Brucella abortus* and *Staphylococcus aureus* in a child	Case report	12 Y/M	Turkey
Santini et al. [10]	A case of brain abscess due to *Brucella melitensis*	Case report	41 Y/F	Italy
Güven et al. [11]	Pituitary abscess secondary to neurobrucellosis. Case illustration	Case report	30 Y/F	Turkey
Seidel et al. [12]	Neurobrucellosis presenting as leukoencephalopathy: the role of cytotoxic T lymphocytes	Case report	65 Y/M	Iran
Solaroglu et al. [13]	Solitary extra-axial posterior fossa abscess due to neurobrucellosis	Case report	23 Y/M	Turkey
Kizilkilic et al. [14]	Successful medical treatment of intracranial abscess caused by *Brucella* spp.	Case report	30 Y/M	Turkey
Koc [15]	Brucellar brain abscess and bilateral arachnoid cysts, unilaterally complicated by subdural haematoma	Case report	70 Y/M	Turkey
Keihani-Douste et al. [16]	A quadriplegic child with multiple brain abscesses: case report of neurobrucellosis	Case report	12 Y/M	Iran
Guvenc et al. [17]	Brucellosis in a child complicated with multiple brain abscesses	Case report	4 Y/M	Turkey
Calik et al. [18]	Severe neurobrucellosis in a young infant	Case report	0.3 Y/F	Turkey
Yilmaz et al. [19]	A rare cause of seizures: brucellar brain abscess	Letter to the editor	8 Y/F	Turkey
Zhang et al. [20]	Treatment of a subdural empyema complicated by intracerebral abscess due to *Brucella* infection	Case report	55 Y/M	China
Turkoglu et al. [21]	Vasculitis and neurobrucellosis: evaluation of nine cases using radiologic findings	Case series	56 Y/F, 33 Y/F, 37 Y/M, 25 Y/M, 53 Y/M, 65 Y/F, 50 Y/F, 77 Y/F, 44 Y/M	Turkey
Chiappe-Gonzalez et al. [22]	A case of cerebral granuloma and optic papillitis due to *Brucella* sp.	Case report	24 Y/F	Peru
Yamout et al. [23]	Neurobrucellosis presenting as longitudinally extensive transverse myelitis: a case report and review of the literature	Case report	44 Y/M	Lebanon
Baghi et al. [24]	Brucellar cervical epidural abscess - a rare cause of neck pain	Case report	22 Y/M	Qatar
Mehta [25]	Neurobrucellosis presented with hemorrhagic stroke: a rare case report	Case report	28 Y/F	India
Qasim et al. [26]	A case of seronegative pediatric neurobrucellosis presenting with ataxia	Case report	9 Y/F	Saudi Arabia
Rozis et al. [27]	Chronic undiagnosed brucellosis presenting as sciatica	Case report	51 Y/M	Greece
Akbulut et al. [28]	Subdural empyema caused by brucellosis: a case report and review of the literature	Case report	27 Y/M	Turkey
De la Peña-Sosa et al. [29]	Pituitary abscess causing panhypopituitarism in a patient with neurobrucellosis: case report	Case report	55 Y/M	Mexico
Zhang et al. [30]	Case report of neurobrucellosis: a rare complication and neuroimaging findings of a common disease	Case series	48 Y/M, 33 Y/M, 38 Y/M, 26 Y/M	China

Data Extraction and Analysis

Variables included patient demographics (age, sex, and country), symptoms, signs, MRI findings, brain region involvement, history of farming or animal husbandry, management, disease dissemination, species of the infectious organism, CSF findings, and serological findings. These were recorded in predesigned tables using Microsoft Word 365 (Microsoft Corporation, Redmond, WA, USA). Four independent reviewers (MM, FI, MS, and HK) screened, scrutinized, and extracted the data under the above-mentioned variables. The data were then verified for errors and accuracy. Clinical trends and patient characteristics were compiled. CT, MRI, and other diagnostic findings were classified. All extracted data were imported into IBM SPSS Statistics for Windows, version 24.0 (released 2016; IBM Corp., Armonk, NY, USA) for statistical analysis. Descriptive statistics were used to summarize the data. Tables were included to present the data in a structured manner.

Scope and Focus of the Review

This narrative review predominantly highlights intracranial manifestations; however, a limited number of patients showed spinal involvement, including transverse myelitis, cervical epidural abscess, and radiculopathy-related presentations. This highlights the heterogeneous neuraxial involvement of neurobrucellosis and emphasizes the need for comprehensive CNS evaluation in suspected cases. Therefore, the focus was to assess data that provided reproducible results (Table 1).

Results

The majority of patients in these studies were male, 22 (64.70%). The mean age of the cohort was 35.74 ± 19.45 years. The majority of cases were from Turkey (n = 18), China (n = 5), and Iran and Mexico (n = 2 each).

The most common presenting symptoms were headache in 23 (67.64%) patients, fever in 16 (47.05%), vomiting in 11 (32.35%), and fatigue in four (11.76%) (Table 2).

**Table 2:** Distribution of symptoms in the study population (N = 34)

Symptomatology	Number of patients (n)	Percentage (n/N)
Headache	23	67.64%
Fever	16	47.05%
Vomiting	11	32.35%
Fatigue	4	11.76%

**Figure 1:**
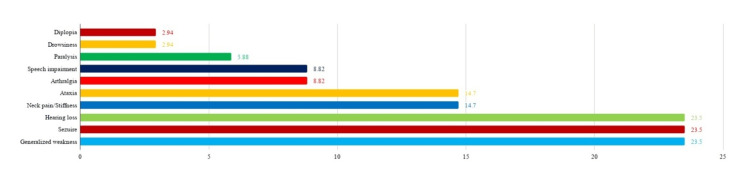
Frequency and percentage occurrence (%) of clinical signs in patients with neurobrucellosis (N = 34) This figure was created using Microsoft Word and Microsoft Excel (Microsoft Corporation, Redmond, WA, USA).

Among the neurological symptoms, generalized weakness, seizures, and hearing loss were noted in eight patients (23.52% each). Other symptoms included neck pain/stiffness in five (14.70%), ataxia in five (14.70%), arthralgia in three (8.82%), speech impairment in three (8.82%), paralysis in two (5.88%), and drowsiness and diplopia in one (2.94%) each (Figure 1).

The most common imaging modality used in patients was MRI in 32 (94.11%), followed by CT scan in 10 (29.41%). The radiological findings on MRI included hypointense, heterogeneous, and multiloculated lesions in 11 (32.35%) patients on T1-weighted images and diffuse hyperintense lesions on T2-weighted images in eight (23.52%) patients. Other findings included meningeal enhancement, ring enhancement, and fusiform enhancement in one patient (2.94%) each (Table 3).

**Table 3:** MRI brain findings among patients with neurobrucellosis (N = 34)

MRI findings	Number of patients (n = 34)	Percentage occurrence (n/N)
Hypointense, heterogeneous, and multiloculated lesions (T1)	11	32.35%
Diffuse hyperintense lesions (T2)	8	23.52%
Meningeal enhancement	1	2.94%
Ring-type enhancement	1	2.94%
Frontal lobe enhancement (fusiform/circular)	1	2.94%

The frontal lobe was the most frequently affected brain region in 11 (32.35%) patients, followed by the occipital and temporal lobes in five (14.70%) patients each, and the parietal lobe in four (11.76%) patients (Table 4).

**Table 4:** Site of brain involvement in neurobrucellosis (N = 34)

Site of involvement	Number of patients (n = 34)	Percentage (n/N)
Frontal lobe	11	32.35%
Temporal lobe	5	14.70%
Occipital lobe	5	14.70%
Parietal lobe	4	11.76%

On performing serological investigations, 30 (88.23%) patients tested positive on the serum agglutination test, 13 (38.23%) on the Rose Bengal test, and eight (23.52%) on the Coombs test (Table 5).

**Table 5:** Serological investigations used among patients with neurobrucellosis (N = 34)

Serology positivity	Number of patients (n = 34)	Percentage (n/N)
Serum agglutination test	30	88.23%
Rose Bengal test	13	38.23%
Coombs test	8	23.52%

CSF analysis was performed in 23 (67.6%) patients, revealing elevated protein in 13 (38.23%), leukocytosis in 10 (29.41%), and growth of *Brucella *on culture in four (11.76%) patients (Table 6).

**Table 6:** Findings of CSF analysis in patients with neurobrucellosis (N = 34)

CSF results	Number of patients (n = 34)	Percentage occurrence (n/N)
Raised protein levels	13	38.23%
Increased WBC count	10	29.41%
Positive CSF culture	4	11.76%

In total, 24 cultures (70.58%) were performed, including blood, abscess, CSF, and urine cultures. *Brucella *was detected in blood cultures of seven (20.58%) patients, in CSF cultures of four (11.76%) patients, and in abscess fluid cultures of three (8.82%) patients (Table 7).

**Table 7:** Types of cultures performed in patients with neurobrucellosis (N = 34)

Culture	Number of patients (n = 34)	Percentage occurrence (n/N)
Total cultures performed (abscess, blood, CSF, and urine)	24	70.58%
Negative culture results	10	29.41%
Blood cultures positive	7	20.58%
CSF cultures positive	4	11.76%
Abscess cultures positive	3	8.82%

The species identified were *Brucella melitensis *in six (17.64%) patients, followed by *Brucella abortus *in two (5.88%) patients (Table 8).

**Table 8:** Species of Brucella identified in included patients (N = 34)

Species identified	Number of patients (n)	Percentage occurrence (n/N)
Unspecified Brucella species	7	20.58%
Brucella melitensis	6	17.64%
Brucella abortus	2	5.88%

In total, 21 (61.76%) patients received medical/conservative treatment, while 12 (35.29%) patients underwent combined surgical and medical management (Table 9). 

**Table 9:** Management strategies used in included cases (N = 34)

Management	Number of patients (n)	Percentage occurrence (n/N)
Medical/conservative	21	61.76%
Surgical + medical	12	35.29%

The most commonly administered empirical drug was ceftriaxone in 10 (29.41%) patients, followed by streptomycin in three (8.82%) patients. Tetracycline, ampicillin, ciprofloxacin, and vancomycin were each used in two patients (5.88%) (Table 10).

**Table 10:** Empirical antimicrobial treatment used in included cases (N = 34)

Empirical treatment	Number of patients (n)	Percentage occurrence (n/N)
Ceftriaxone	10	29.41%
Streptomycin	3	8.82%
Vancomycin	2	5.88%
Ciprofloxacin	2	5.88%
Tetracycline	2	5.88%
Ampicillin	2	5.88%
Meropenem	1	2.94%
Methylprednisolone	1	2.94%

**Figure 2:**
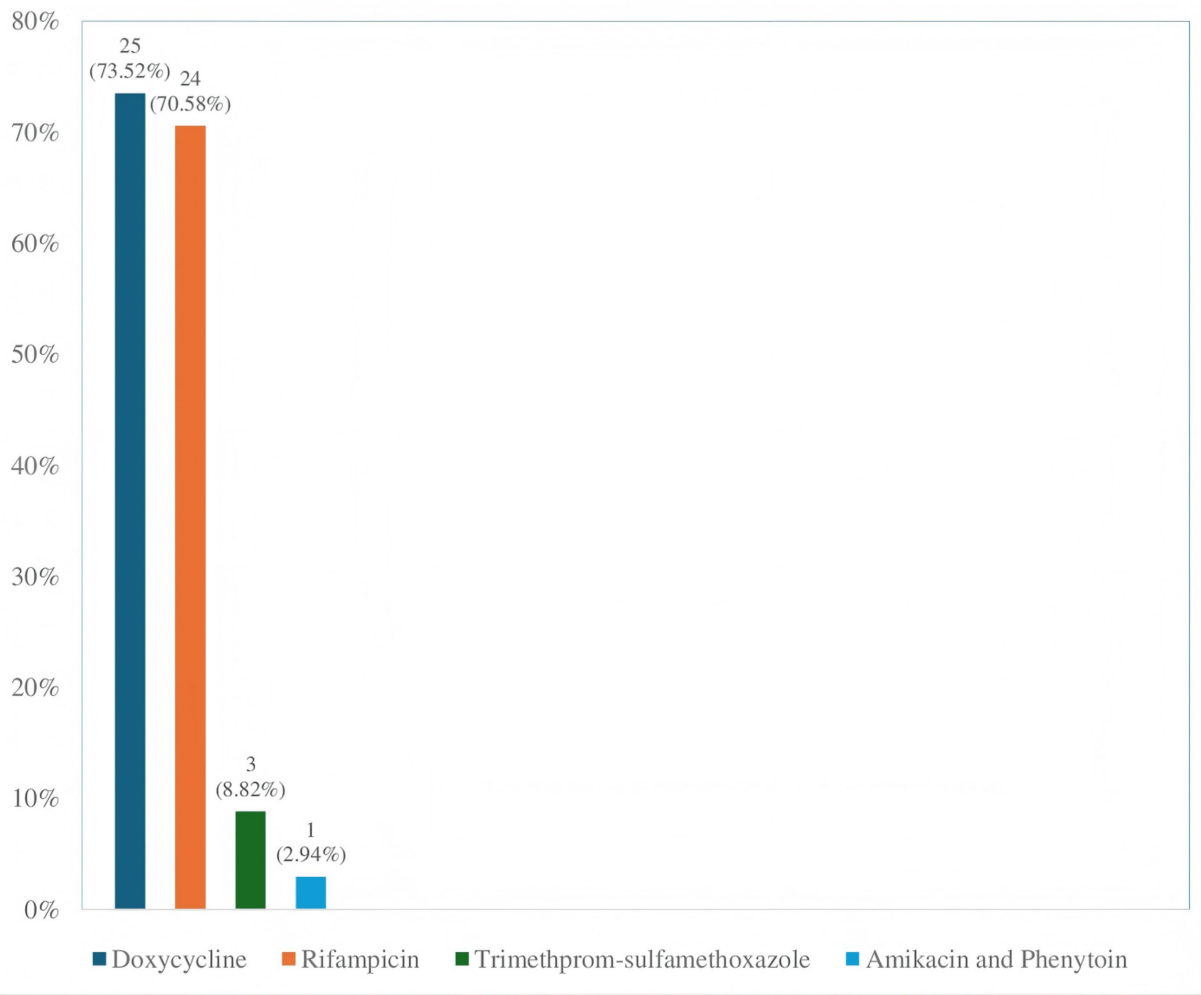
Definitive treatment used among patients with neurobrucellosis (N = 34) This figure was created using Microsoft Word and Microsoft Excel (Microsoft Corporation, Redmond, WA, USA).

Doxycycline was the most commonly used drug in 25 (73.52%) patients, followed by rifampicin in 24 (70.58%), trimethoprim-sulfamethoxazole in three (8.82%), and amikacin and phenytoin in one (2.94%) patient each (Figure 2).

**Figure 3:**
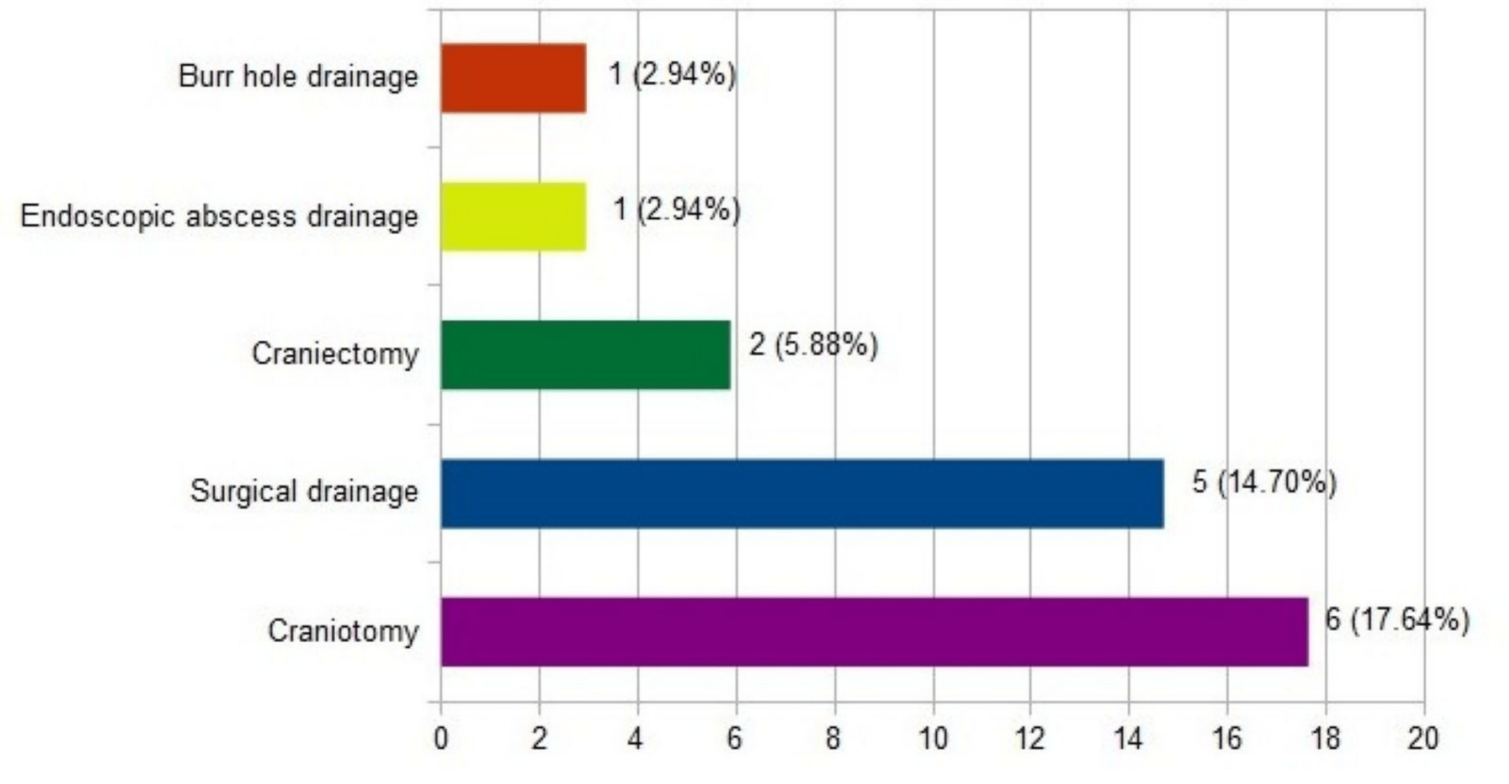
Surgical interventions performed in patients with neurobrucellosis (N = 34) This figure was created using Microsoft Word and Microsoft Excel (Microsoft Corporation, Redmond, WA, USA).

The most common surgical intervention was craniotomy in six (17.64%) patients, followed by surgical drainage in five (14.70%) patients and craniectomy in two (5.88%) patients, followed by endoscopic abscess drainage and burr hole drainage (Figure 3).

Regarding clinical outcomes, complete resolution was observed in 23 (67.64%) patients, while relapse occurred in five (14.70%) patients, and one (2.94%) patient died (Table 11).

**Table 11:** Clinical outcomes after treatment in patients with neurobrucellosis (N = 34)

Clinical status	Number of patients (n = 34)	Percentage occurrence (n/N)
Complete resolution	23	67.64%
Relapse	5	14.70%
Death	1	2.94%

Among patients who received medical treatment alone, complete recovery was seen in 17 (50%) patients, while two (5.88%) patients experienced relapse and one (2.94%) patient died. Among patients who received combined surgical and medical treatment, six (17.64%) patients recovered and four (11.7%) patients had a relapse. No deaths were reported in the latter group. The average follow-up duration was 6 ± 7 months.

Discussion

The global incidence of human brucellosis is 1.6-2.1 million new cases per year, which is three to four times higher than the previous estimate of 500,000 new cases per year [31]. Brucellosis remains endemic in the Mediterranean Basin, the Middle East, South Asia, and Latin America, paralleling livestock infection rates and food-handling practices [32]. In this study, most cases were from Turkey, corresponding to known endemic regions, as Turkey geographically spans Europe, Asia, and the Mediterranean belt. In addition to endemicity, other factors that affect the incidence of brucellosis include veterinary control procedures, food-handling safety, clinician awareness, and diagnostic availability [32]. The majority of patients in these studies were male, and the mean age was 35.74 ± 19.45 years, similar to another study reporting mean ages between 35 and 40 years with male predominance [33,34]. In our study, eight patients (23.52%) had a previous history of raw milk ingestion; one of these cases also suggests vertical transmission through breast milk.

Pathophysiology and Mechanisms of CNS Involvement

Although the exact mechanisms by which *Brucella *species enter the central nervous system are unknown, a combination of bacterial, immunological, and vascular pathways appears to be involved. Through direct contact, inhalation of aerosols, or consumption of contaminated animal products, *Brucella *enters the human body and spreads via the bloodstream and reticuloendothelial system. Spread to the CNS occurs via either trans-endothelial migration or infected phagocytes [32]. Within the CNS, the bacteria may persist intracellularly, leading to chronic inflammation [35]. This may manifest as meningitis, meningoencephalitis, parenchymal involvement, myelitis, or spinal radiculopathy [2]. Recent evidence indicates that *Brucella *spp. evade host immunity through modulation of intracellular trafficking and inhibition of phagosome-lysosome fusion, enabling prolonged survival within macrophages and glial cells. This intracellular persistence promotes chronic infection and relapse while triggering sustained neuroinflammatory cascades that contribute to CNS damage [36]. Recognizing these mechanisms underpins rational diagnostic approaches (e.g., CSF and MRI) and the selection of antibiotics with intracellular activity and blood-brain barrier penetration.

Clinical Manifestations and Disease Spectrum

CNS brucellosis presents as meningitis, meningoencephalitis, myelitis, cranial neuropathies, and intracranial hypertension [35]. In our study, headache was the most common symptom in 23 patients (67.64%), followed by fever in 16 (47.05%) and vomiting in 11 (32.35%), while fatigue was reported in four (11.76%) patients. Neurological findings included generalized weakness, seizures, and hearing loss in eight (23.50%) cases; neck stiffness and ataxia in five (14.70%); and paralysis in two (5.88%) patients. These findings suggest that clinical patterns primarily fall into focal neurological syndromes and meningitis-predominant syndromes. Recent studies have shown similar trends among 21 Chinese patients, including fever in 14 (66.7%), fatigue in 12 (57.1%), hearing loss in 10 (47.5%), and limb weakness in 11 (52.4%) patients [34]. Similarly, fever in 49 (91%) and headache in 47 (87%) were the most common symptoms in 54 Iranian adults with neurobrucellosis, whereas seizures occurred in six (11%) patients [33]. The average time to diagnosis was 8.83 months (265 days) in this study.

Neurobrucellosis can mimic demyelinating or neoplastic disorders, as demonstrated by Yazdi et al., who documented diffuse involvement of the cerebellum, brainstem, spinal cord, and cauda equina [6]. Due to overlapping symptoms and limited access to advanced diagnostics such as PCR, it is frequently misdiagnosed as viral meningitis or tuberculosis in endemic areas [7]. In this study, patients were initially diagnosed with meningitis in two (5.88%) cases, tuberculous meningitis in two (5.88%), ear infection in one (2.94%), subdural hematoma in one (2.94%), meningoencephalitis in one (2.94%), and ependymoma in one (2.94%) case.

Radiological and Laboratory Diagnosis

MRI remains the most sensitive tool for identifying CNS involvement in brucellosis. In this study, MRI was performed in 32 cases (94%), with 11 (32%) showing hypointense, heterogeneous, and multiloculated lesions on T1, while eight (23.52%) showed hyperintense lesions on T2. Other findings included meningeal enhancement and ring-type enhancement (3% each) (Table 3). Comparatively, other studies report MRI findings such as myelitic changes, granulomatous lesions, and leptomeningeal enhancement as characteristic imaging features [1]. The most common intracranial location was the frontal lobe, seen in 11 (32.35%) cases, consistent with the study by Al-Sous et al. [37].

Among biochemical investigations, 30 (88.23%) patients had a positive serum agglutination test, highlighting its diagnostic utility. Thirteen (38.23%) tested positive on the Rose Bengal test and eight (23.52%) on the Coombs test, demonstrating the value of serological testing (Table 5). These positivity rates closely align with findings from Zhuang et al., who also identified the serum agglutination test (94.74% positive) as the most reliable diagnostic tool for neurobrucellosis [34]. Among 34 patients, 23 (67.6%) underwent CSF analysis; 13 (38.2%) showed elevated protein, 10 (29.41%) had leukocytosis, and four (11.76%) had culture positivity. These findings are generally consistent with those of Naderi et al., who reported elevated CSF protein (72%) and other abnormalities in 92% of cases [33].

Therapeutic Approaches and Antimicrobial Regimens

CNS brucellosis requires prolonged combination antimicrobial therapy with drugs that have good CNS penetration and intracellular activity [38]. The most accepted regimens combine doxycycline and rifampicin, often with a third agent such as ceftriaxone, an aminoglycoside, or trimethoprim-sulfamethoxazole [38]. Treatment usually extends over three to six months or longer in chronic or relapsing cases, and surgical intervention is sometimes necessary for abscesses or mass lesions [38]. In our study, ceftriaxone was the most common empiric drug in 10 (29.4%) cases, followed by streptomycin in three (8.8%), while doxycycline in 25 (73.5%) and rifampicin in 24 (70.5%) were the mainstays of definitive therapy (Figure 2). Trimethoprim-sulfamethoxazole was used in three (8.8%) cases. These findings are consistent with previous reports in which multidrug regimens centered on doxycycline and rifampicin remain the preferred treatment, frequently supplemented with ceftriaxone or aminoglycosides for severe CNS involvement [4,38]. Prolonged therapy is required due to *Brucella*’s intracellular persistence in macrophages and glial cells [32]. However, drug toxicity, poor adherence, and limited access to parenteral agents in resource-limited settings can affect outcomes.

Neurosurgical Management and Indications

Neurosurgical intervention in CNS brucellosis is reserved for complications such as intracerebral abscess, hydrocephalus, or spinal cord compression [38]. In our series, surgical interventions included craniotomy in six (17.64%) cases, surgical drainage in five (14.70%), craniectomy in two (5.88%), and endoscopic and burr-hole drainage in single cases (2.94% each) (Figure 3). Most patients respond to medical management; however, timely neurosurgical intervention remains essential to prevent irreversible neurological damage.

Outcome and Prognosis

Early diagnosis, appropriate antimicrobial duration, and management of complications are critical factors in clinical outcomes for CNS brucellosis. In our study, the average follow-up duration was 6 ± 7 months, and complete recovery was observed in 23 patients (67.64%), relapse in five (14.70%), and death in one patient (2.94%), while outcomes were not reported in five (14.70%) cases. Among 21 (61.76%) patients treated medically, 17 (50%) recovered, two (5.88%) relapsed, and one (2.94%) died, while in the combined surgical-medical group (12 patients, 35.29%), six (17.64%) recovered and four (11.76%) experienced relapse, with no mortality (Table 11). Similar trends were noted in the meta-analysis by Tajerian et al., where relapse occurred in six patients (2.4%) treated medically [38]. These results are generally in line with larger series from endemic regions [38], which reported six (2.4%) relapses and approximately 73% recovery, and observational studies such as Patra et al. [4], which reported two (1%) mortalities. Relapses are generally attributed to inadequate treatment duration or poor adherence, reinforcing the importance of prolonged therapy and close follow-up.

**Figure 4:**
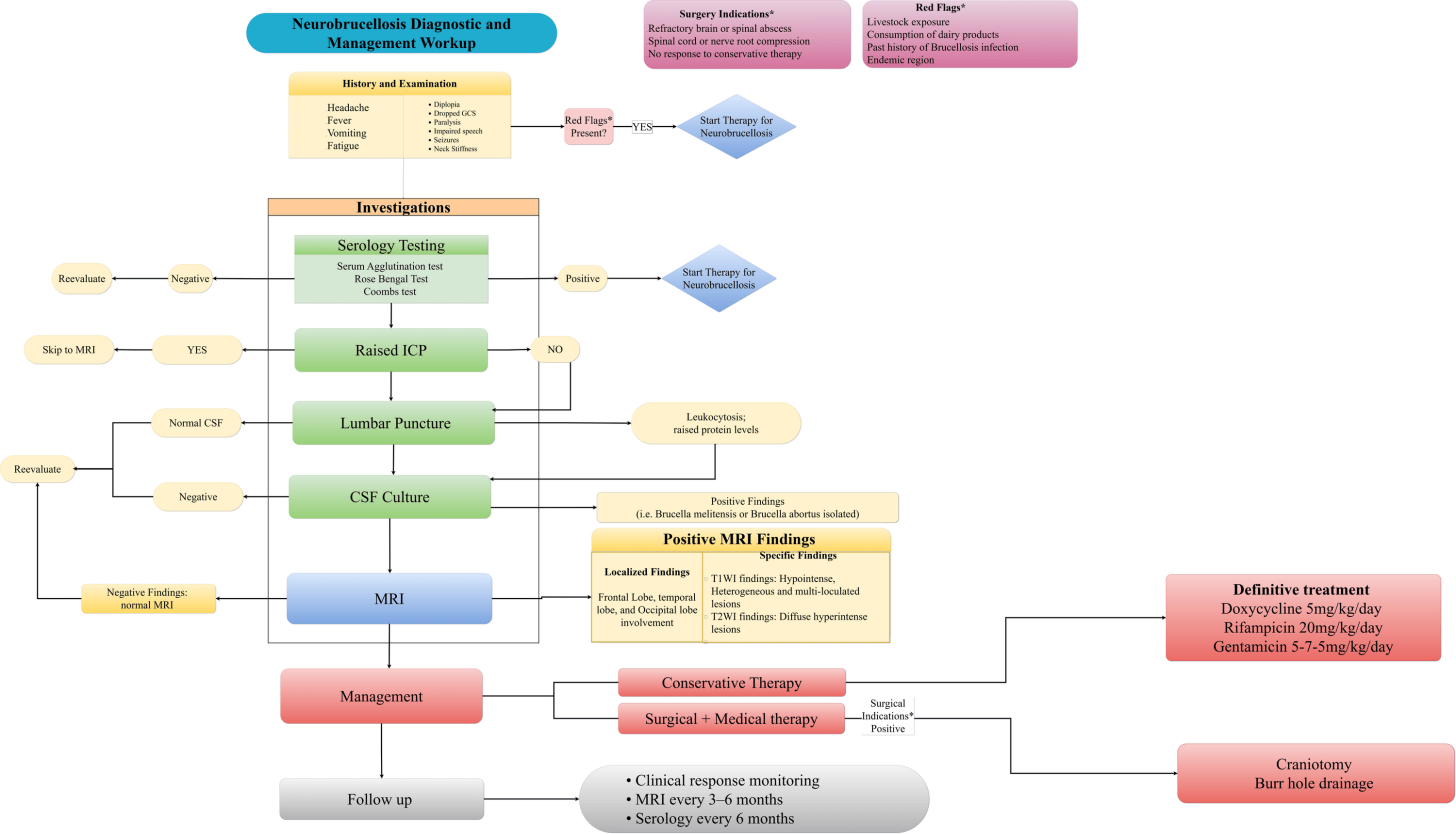
Author-proposed schematic flowchart for the diagnosis and management of neurobrucellosis GCS, Glasgow Coma Scale; ICP, intracranial pressure; T1WI, T1-weighted imaging; T2WI, T2-weighted imaging This figure was created using diagrams.net (draw.io; JGraph Ltd., Northampton, UK).

An author-proposed diagnostic and management algorithm has been designed to guide the evaluation and management of patients presenting with neurobrucellosis (Figure 4).

Clinical Recommendation

Neurobrucellosis should be considered in the differential diagnosis of chronic or unexplained central nervous system pathology, particularly in patients from endemic regions or those with risk factors such as livestock exposure or consumption of unpasteurized dairy products. In resource-limited settings, timely initiation of appropriate and prolonged combination antibiotic therapy is essential to prevent neurological complications and disability. Standardized guidelines should be implemented in endemic regions to guide diagnosis and management.

Limitations

We used two databases, PubMed and Google Scholar. The sample size of included studies was limited. The predominance of cases was from Turkey, an endemic region. As a result, neurobrucellosis from non-endemic regions is underrepresented, which may limit generalizability. Most signs and symptoms were nonspecific neurological features; therefore, we could not propose a definitive diagnostic protocol.

## Conclusions

Neurobrucellosis, though based on limited data, shows male predominance and often presents with nonspecific prodromal symptoms, making early diagnosis challenging. Imaging commonly reveals frontal lobe involvement with characteristic hypointense multiloculated lesions on T1-weighted MRI and hyperintense signals on T2-weighted MRI. Diagnosis relies on positive serological testing, particularly serum agglutination tests, in conjunction with clinical and radiological findings. Optimal outcomes require a tailored multimodal management approach incorporating appropriate antimicrobial therapy with surgical intervention when indicated.
